# Looking into the future

**DOI:** 10.7554/eLife.03146

**Published:** 2014-05-28

**Authors:** Vincent D Costa, Bruno B Averbeck

**Affiliations:** 1 **Vincent D Costa** is in the Laboratory of Neuropsychology, National Institute of Mental Health, Maryland, United Statesvincent.costa@nih.gov; 2 **Bruno B Averbeck** is in the Laboratory of Neuropsychology, National Institute of Mental Health, Maryland, United States

**Keywords:** non-human primate, oculomotor system, population decoding, Bayesian inference, neural processing, other

## Abstract

Eye tracking experiments show that neurons respond rapidly to eye movements, allowing our view of the world to remain stable.

**Related research article** Graf ABA, Andersen RA. 2014. Inferring eye position from populations of lateral intraparietal neurons. *eLife* **3**:e02813. doi: 10.7554/eLife.02813

**Image** The direction of eye movements can be predicted by looking at the firing patterns of neuron populations in the parietal cortex

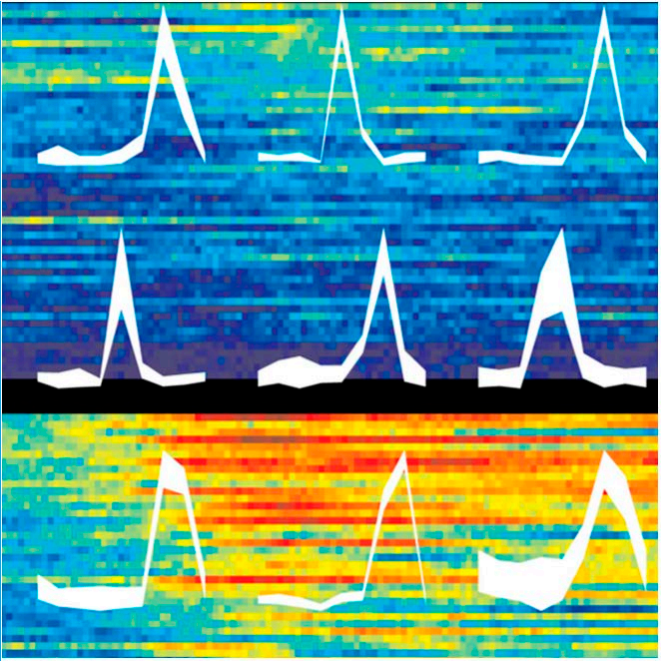

In daily life, we carry out numerous tasks that require a high level of visual awareness. For example, we can reach for a cup of coffee without looking directly at it, we can walk down the street without bumping into other people, and we can drive a car without thinking about it. Because we can perform these tasks so easily, it seems as though our brain can work out the positions of objects with very little effort. In fact, to do this the brain must process a lot of complex information.

We see things because receptors on the retina are excited by photons of light, and our brain represents this information in the visual cortex. However, if we move our eyes, receptors in a different part of the retina are excited, and the new information is stored in a different part of the visual cortex—but we still know that the objects we can see are in the same place. How, then, does the brain ensure that we can continue to perform tasks that require us to know exactly where objects are, while all these changes are going on?

It has been proposed that a mechanism called gain field coding makes this possible (Zipser and Andersen, 1988). This is a form of population coding: that is, it involves many neurons firing in response to a given visual image, rather than just one neuron firing. Neurons with gain field coding represent both the location of objects on the retina and the angle of gaze (i.e., where we are looking in space). From this information, computational models have shown that the location of objects in space can be calculated (Pouget and Sejnowski, 1997). However, for this mechanism to work effectively, the angle of gaze must be reliably represented and rapidly updated after an eye movement. Now, in *eLife*, Arnulf Graf and Richard Andersen of the California Institute of Technology show that the neural population code for eye movements and eye position in a region of the brain called the parietal cortex is accurate, and is updated rapidly when eye movements are planned and executed (Graf and Andersen, 2014).

To demonstrate this, monkeys carried out a task where they had to make saccades—rapid movements of the eyes (Figure 1). At the same time, the response of a population of neurons in an area of the parietal cortex called LIP (Lateral-Intra-Parietal) was recorded. Area LIP has previously been associated with behaviour related to eye movements (Gnadt and Andersen, 1988).

**Figure 1.**
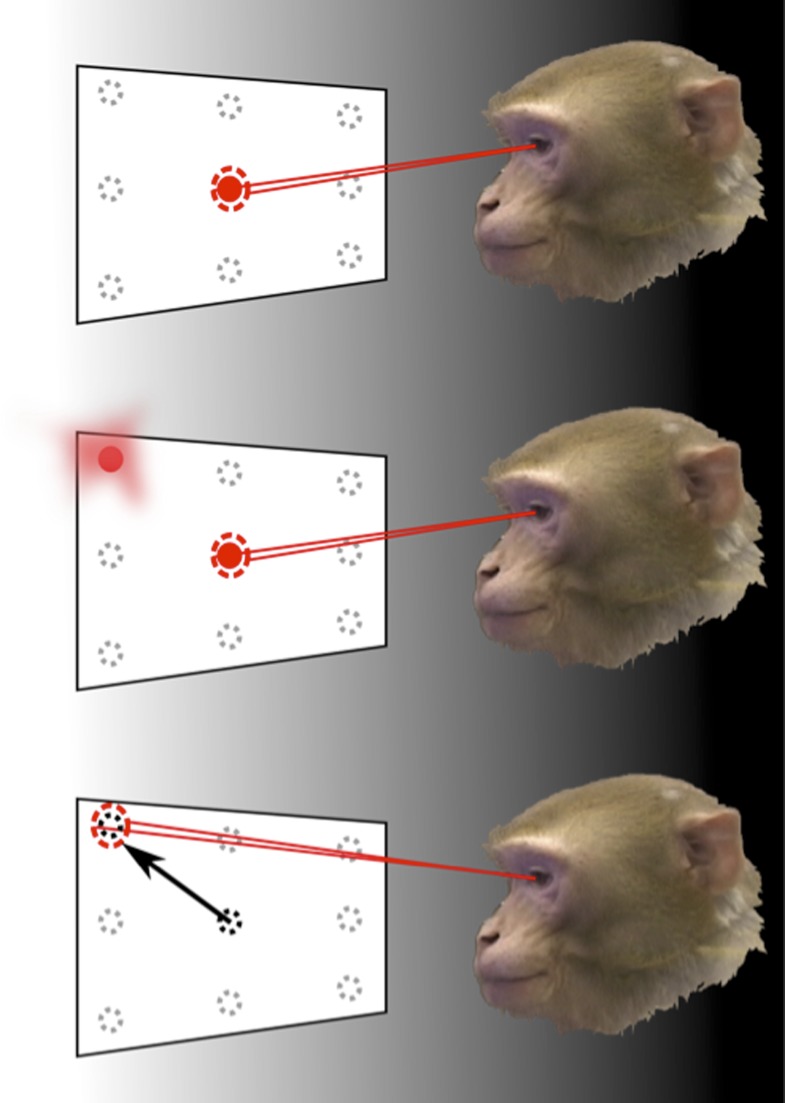
How the brain represents information about the locations of objects can be revealed through memory-guided saccade tasks, performed in the dark. To find out how the neurons in area LIP of the parietal cortex respond to eye movements and eye position, Graf and Andersen trained monkeys to rapidly move (saccade) their eyes to the remembered location of a target, while the response of their neurons was monitored. The monkey initially fixated on one of nine target positions (top). Then, one of the surrounding target locations was flashed before disappearing (middle). The animals had to remember the target location for a short period of time and then move their eyes to look at this location when the fixation point disappeared (bottom). The experiments were carried out in the dark to eliminate the possibility that the recorded neural response was caused by any other visual information.

The task performed by the monkeys had to be carefully designed to eliminate a range of possible confounding factors. If the targets were visible when eye movements were made towards them, any detected neural activity may have been representing the locations of those targets, rather than the eye movements. Therefore, eye movements were made in darkness and the planned movement had to be remembered by the monkey. The task also separated the direction of the eye movement from the position of the eye before and after the movement. This allowed Graf and Andersen to examine, unambiguously, whether information in the neural population code was representing either—or both—current and future eye position signals.

To determine whether the population code in the parietal cortex contained information about eye position and eye movements, Graf and Andersen used statistical models to analyse the neural activity and estimate these two variables at specific points in time. If these variables can be read out by a decoding analysis, this information will also be available in the brain and will, therefore, also be able to drive behaviour.

Graf and Andersen found that population coding of the initial eye position was represented well throughout the eye movement. In contrast, the coding of the final eye position began after the target location was flashed—at the point in the task when the animals were told where to move their eyes—and peaked following the completion of the eye movement. This finding builds on existing evidence that eye position can be decoded from area LIP before and after a saccade to a visual target (Morris et al., 2013).

Contrary to recent suggestions by Xu et al. (Xu et al., 2012), Graf and Andersen show that population coding of the post saccadic eye position signal was updated quickly after the saccade target was shown. There are two possible reasons for the discrepancies between these studies. First, Xu et al. assumed that eye position information could be characterised by a number called the gain field index. Xu et al. also only examined single neurons. Even though individual neurons represent fixation location and eye movements, different combinations of eye positions and target locations can cause some neurons to respond in the same way. However, looking at a population of neurons removes this ambiguity so that it is clear what the neurons are actually responding to—and this can be achieved with decoding analyses (Georgopoulos et al., 1986).

It is well recognised that the brain computes and produces behaviour on the basis of distributed representations of neural activity, where patterns of activity across many neurons represent one action, and each neuron is involved in more than one action (Fetz, 1992; Rigotti et al., 2013). Distributed representations can be non-intuitive—but they are the way the brain represents and processes information. Graf and Anderson illustrate the use of decoding to extract information from a distributed representation across a population of neurons, and show that this approach can resolve debates about neural coding. This study also points to the importance of recording from large neural populations when investigating how complex tasks are performed, so the space in which population coding applies is fully explored.
